# T‐cell‐derived cytokines enhance the antigen‐presenting capacity of human neutrophils

**DOI:** 10.1002/eji.201848057

**Published:** 2019-08-06

**Authors:** Nazanin Samadi, Dominika Polak, Claudia Kitzmüller, Peter Steinberger, Gerhard J. Zlabinger, Beatrice Jahn‐Schmid, Barbara Bohle

**Affiliations:** ^1^ Department of Pathophysiology and Allergy Research Medical University of Vienna Vienna Austria; ^2^ Institute of Immunology, Center of Pathophysiology, Infectiology and Immunology Medical University of Vienna Vienna Austria

**Keywords:** Allergen‐specific T cells, Allergy, Neutrophils, Th1, Th2

## Abstract

Activated allergen‐specific Th2 and Th1 cells release cytokines that transform neutrophils into functional APCs characterized by the expression of HLA‐DR and CD58 as well as enhanced survival and antigen uptake, irrespectively of the presence of IL‐10, which reduces allergen uptake by neutrophils.

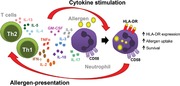

Allergen exposure of allergic individuals induces immediate reactions caused by histamine and other mediators released from sensitized mast cells as a consequence of IgE cross‐linking. After 3–48 h, cell‐mediated late‐phase reactions may follow, characterized by early accumulation of neutrophils together with allergen‐specific CD4^+^ T‐cells and subsequent infiltration of eosinophils 1, 2. Up to now, neutrophils were regarded to contribute to inflammation by released mediators. However, we detected HLA‐DR‐positive neutrophils in cutaneous allergic late‐phase reactions in vivo and demonstrated in vitro that in the presence of a cocktail of GM‐CSF, IFN‐γ, and IL‐3, neutrophils internalized, processed, and presented allergen via HLA‐DR resulting in proliferation and cytokine production of specific T cells 3. In turn, anti‐CD3‐activated CD4^+^ and particularly CD8^+^ T cells have been shown to modulate apoptosis and activation of neutrophils 4, 5. As allergen‐stimulated CD4^+^ T cells release several cytokines in addition to GM‐CSF, IFN‐γ, and IL‐3, we sought to assess whether key cytokines of allergen‐specific Th2‐ and Th1‐cells affect the antigen‐presenting activity of neutrophils.

To estimate the amount of cytokines released by allergen‐specific T cells, we specifically stimulated 16 clones from different allergic patients and determined the major Th2‐ and Th1‐like cytokines IL‐4, IL‐5, IL‐13, IL‐2, and IFN‐γ, GM‐CSF, IL‐3, the inflammatory cytokines TNF‐α and IL‐6, the immunosuppressive cytokine IL‐10, as well as IL‐17, which contributes to the recruitment of neutrophils. We classified Th1‐ or Th2‐cells according to IL‐4/IFN‐γ ratios and found extremely heterogeneous cytokine patterns released by either subset (Supporting Information Table 1). In addition to the anticipated high levels of IL‐4, IL‐5, and IL‐13, several Th2‐clones produced remarkable levels of IL‐2, IL‐3, IL‐10, and/or IL‐17. In addition to IFN‐γ, some Th1‐clones produced IL‐3 and IL‐10. With the exception of very few Th2‐clones, both subsets synthesized considerable levels of GM‐CSF, TNF‐α, and IL‐6.

To assess the expression of HLA‐DR, neutrophils from eight birch pollen‐allergic individuals were cultured in medium or stimulated with recombinant IL‐2, IL‐3, IL‐4, IL‐5, IL‐6, IL‐10, IL‐13, IL‐17, IFN‐γ, TNF‐α, or GM‐CSF. In accordance with neutrophils from nonallergic individuals 4, 5, HLA‐DR was upregulated by GM‐CSF, IFN‐γ, and high levels of IL‐3 (Supporting Information Fig. 1A). However, even high levels (10 ng/mL) of the remaining cytokines had no effect (Fig. 1A). Furthermore, none of the cytokines notably enhanced surface expression of CD80 and CD86 nor decreased the expression of CD58, which we recently demonstrated to be involved in functional activation of T‐cells by neutrophils 6 (Fig. 1A). Simultaneous analyses of cell viability revealed that GM‐CSF, IFN‐γ, and high doses of TNF‐α enhanced the percentage of viable neutrophils. Weaker dose‐dependent effects were observed for IL‐2, IL‐3, and interestingly, also for IL‐4 (Supporting Information Fig. 1B). None of the remaining cytokines showed an effect (data not shown).

**Figure 1:**
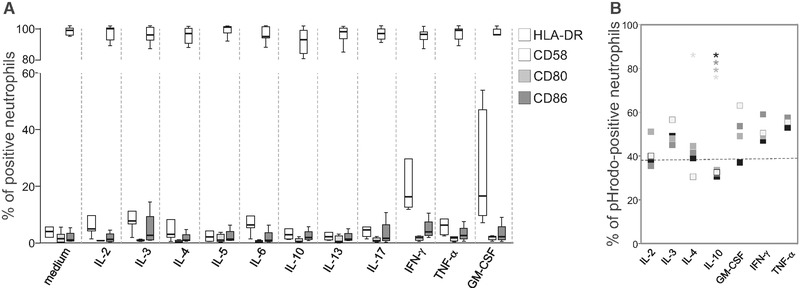
Effects of T‐cell‐derived cytokines on the expression of HLA‐DR, CD80, CD86, and CD58 of neutrophils. (A) The percentage of HLA‐DR^+^, CD80^+^, CD86^+^, and CD58^+^ neutrophils from eight different birch pollen‐allergic individuals after incubation in medium or with 10 ng/mL of recombinant cytokines for 24 h depicted as box plots: boxes show the values within the first and third quartile divided by the median. The whiskers indicate the minimum and maximum of all data. (B) Neutrophils from five different allergic individuals were incubated with pHrodo‐labeled Bos d 5 in the presence of 10 ng (black), 1 ng (dark gray), 0.1 ng (medium gray), and 0.01 ng/mL (light gray) of recombinant cytokines. Squares represent the median values of the percentage of pHrodo^+^ neutrophils, and the dotted line represents the medium control. A single experiment per individual was performed. *p < 0.05, Wilcoxon‐signed ranks test.

To study allergen uptake, we employed β‐lactoglobulin (Bos d 5) labeled with pHrodo™ Red dye that emits fluorescence when phagosomal and granular vesicles fuse and the pH value is reduced. GM‐CSF, IFN‐γ, TNF‐α, IL‐2, and IL‐3 dose dependently enhanced the percentage of pHrodo^+^ neutrophils (Fig. 1B). Notably, even low doses of IL‐10 significantly reduced allergen uptake as did low amounts of IL‐4. No impact of the remaining cytokines was found (data not shown). As several allergen‐stimulated Th2‐ and Th1‐clones produced IL‐10 (Supporting Information Table 1), we wondered whether this immunosuppressive cytokine still diminishes allergen uptake of neutrophils in combination with other cytokines. Therefore, we incubated neutrophils from six birch pollen‐allergic donors with pHrodo‐labeled Bos d 5 in the presence of cytokine cocktails corresponding to the cytokines released by the clones Th2‐1, Th2‐2, Th2‐3, Th1‐1, Th1‐2, or Th1‐3 (Supporting Information Table 1). All cocktails significantly upregulated allergen uptake, however, cocktail Th2‐1, containing very high levels of IL‐10 and no IFN‐γ, and to a lesser extent also cocktail Th2‐3, showed a lower impact than the other cocktails (Fig. 2A). In parallel, all cocktails induced a comparable upregulation of HLA class II molecules (Fig. 2A). Next, we incubated neutrophils with pHrodo‐labeled Bos d 5 in variants of cocktail Th2‐1 lacking GM‐CSF, TNF‐α, or IL‐3 or variants of cocktail Th2‐3 lacking IFN‐γ and TNF‐α, respectively. Figure 2B shows that GM‐CSF counteracted the reduced allergen uptake in the presence of >12 ng/mL of IL‐10 contained in cocktail Th2‐1, whereas IL‐3 and TNF‐α were not effective. In cocktail Th2‐3, per se lacking GM‐CSF and containing 0.4 ng/mL of IL‐10, the presence of either IFN‐γ or TNF‐α was sufficient to rescue allergen uptake (Fig. 2B). To confirm their function as APC, we stimulated neutrophils with the different cytokine cocktails in the absence or presence of the major birch pollen allergen Bet v 1 or Bos d 5, and added them to autologous Bet v 1‐specific T‐cell lines. The proliferative response to Bet v 1 was similar irrespective of the cytokine cocktail used to stimulate neutrophils (Fig. 2C). None of the T‐cell lines proliferated to the negative control Bos d 5. These results indicate that neutrophils may become functional APC in a Th2‐ as well as a Th1‐like cytokine milieu irrespective of the presence of IL‐10.

**Figure 2:**
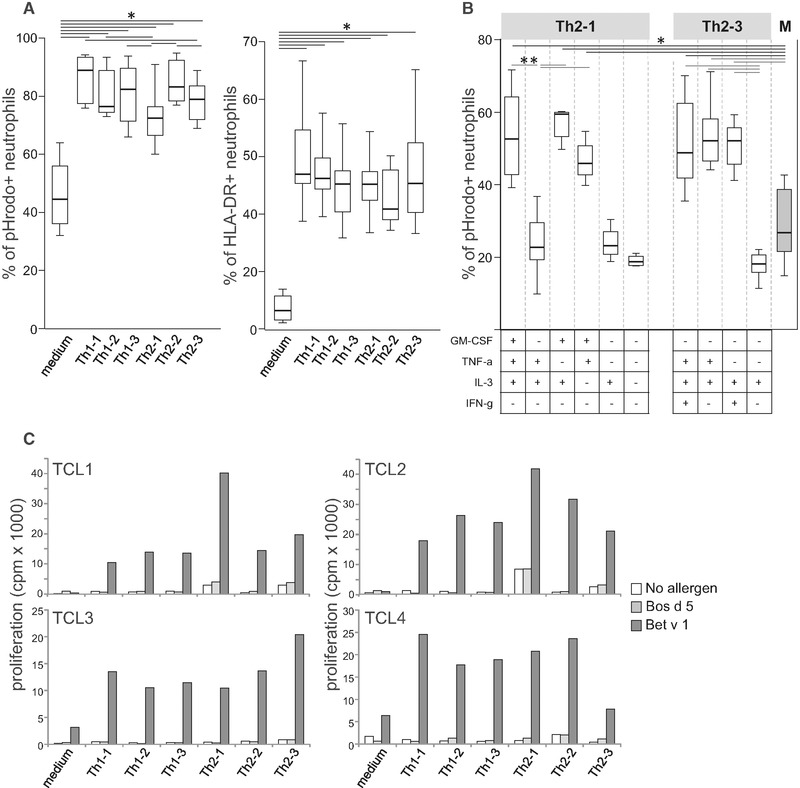
T‐cell‐derived cytokines induce antigen‐presenting activity of neutrophils. (A) The percentage of pHrodo^+^ and HLA‐DR^+^ neutrophils of six allergic individuals after incubation with pHrodo‐labeled Bos d 5 in cytokine mixes as produced by Th1‐ and Th2‐clones (Supporting Information Table 1). (B) The percentage of pHrodo^+^ neutrophils from seven allergic individuals after incubation with pHrodo‐labeled Bos d 5 in the cytokine mixes Th2‐1 and Th2‐3, their variants lacking GM‐CSF, TNF‐α, IL‐3, and IFN‐γ, or medium (M). (A, B) A single experiment per individual was performed. Box plots are shown; the box includes the first and third quartile divided by the median. The whiskers indicate the minimum and maximum of all data. *p < 0.05, Wilcoxon signed‐ranks test. (C) Proliferation of four Bet v 1‐specific T‐cell lines (TCL) in response to neutrophils incubated without or with Bet v 1 or Bos d 5 in the absence or presence of cytokine mixes; cpm, counts per minute; bars show the mean value of duplicates.

In summary, GM‐CSF most potently transformed neutrophils from allergic individuals into functional APC 4, 5, 7. Similar to phagocytosis of bacteria 8, allergen uptake by neutrophils was reduced by IL‐10. However, this inhibitory effect was overruled by the presence of GM‐CSF, IFN‐γ, and TNF‐α and did not diminish their T‐cell‐activating activity. We conclude that cytokines released from both allergen‐specific effector Th1 and Th2‐cells enhance the life‐span and antigen‐presenting activity of neutrophils. In turn, neutrophils activate co‐localized allergen‐specific effector T cells 1, 2 and thereby, amplify allergen‐specific cell‐mediated inflammation 6.

## Author Contributions

N.S., D.P., and C.K. performed the experiments and analyzed the data; P.S. provided antibodies; G.J.Z. helped with cytokine measurements; and N.S., D.P., C.K., B.J.S., and B.B. wrote the manuscript.

## Conflict of interest

The authors declare no commercial or financial conflict of interest.

## Supporting information

Supporting information
