# Supplementary material for: T‐cell‐derived cytokines enhance the antigen‐presenting capacity of human neutrophils
Source: Eur J Immunol. 2019 Aug 6;49(9):1441–3. doi: 10.1002/eji.201848057 (PMC6771661; doi:10.1002/eji.201848057)
Supplement: Supplementary file 1 — Supporting information [file EJI-49-1441-s001.pdf]

European Journal of Immunology

Supporting Information
for
DOI 10.1002/eji.201848057

Nazanin Samadi, Dominika Polak, Claudia Kitzmüller, Peter Steinberger, Gerhard J. Zlabinger, Beatrice Jahn-Schmid and Barbara Bohle

T-cell-derived cytokines enhance the antigen-presenting capacity of human neutrophils

© 2019 The Authors. *European Journal of Immunology* published by WILEY-VCH Verlag GmbH & Co. KGaA, Weinheim.

SUPPORTING INFORMATION

The impact of T cell-derived cytokines on the antigen-presenting capacity of human neutrophils

Nazanin Samadi[*1], Dominika Polak[*1], Claudia Kitzmüller[1], Peter Steinberger[2], Gerhard J. Zlabinger[2], Beatrice Jahn-Schmid[1], and Barbara Bohle[1]

*both authors contributed equally, [1]Department of Pathophysiology and Allergy Research, and [2]Institute of Immunology, Center of Pathophysiology, Infectiology and Immunology, Medical University of Vienna, Vienna, Austria

Running head: Activation of neutrophils by Th2 and Th1 cytokines

This work was supported by the Austrian Science Fund, projects W1248 and SFB F4610, and by the Medical University of Vienna.

Corresponding author:

Barbara Bohle

Department of Pathophysiology and Allergy Research

Medical University of Vienna

Waehringer Guertel 18-20

A-1090 Vienna

Phone: +43 1 40400 51140

Fax: +43 1 40400 61880

Email: barbara.bohle@meduniwien.ac.at

Table 1. Cytokine patterns of allergen-specific CD4$^+$ T cells in response to allergen

TCC	Allergen	IL-2*	IL-3	IL-4	IL-5	IL-13	IL-6	IL-10	IL-17	IFN-γ	TNF-α	GM-CSF
Th2-1**	Amb a 1	480	93	197	>8000	>33600	<6	>12000	<6.0	<6	688	642
Th2-2	Art v 1	514	18	>4500	>8000	>33600	10612	897	38.2	191	12695	3271
Th2-3	Bet v 1	>50000	<6	>4500	6977	13853	601	441	17922	408	>83000	<6.0
Th2-4	Phl p 1	<6	92	218	>8000	>33600	583	21	<6	<6	1118	1321
Th2-5	Amb a 1	63	173	245	1206	15286	>11000	103	<6	21	1591	1204
Th2-6	Amb a 1	374	31	279	1206	20300	52	3714	<6	26	395	488
Th2-7	Bet v 1	22	n.t.	15300	5100	>33600	1950	9200	150	60	2080	9300
Th2-8	BP	3546	170	>4500	236	12923	3683	551	<6	<6	5439	1415
Th1-1	Art v 1	734	224	<6	117	n.t.	778	2179	<6	6648	2420	1032
Th1-2	Bet v 1	12	234	<6	9	304	3147	121	<6	976	2895	3147
Th1-3	Bet v 1	20	<6	<6	80.2	182	>11000	9	<6	130	2344	>11000
Th1-4	Art v 1	382	30.2	<6	138	1194	1866	4234	<6	765	2505	335
Th1-5	BP	n.t.	<6	25	217	n.t.	8919	n.t.	n.t.	11659	8341	492
Th1-6	BP	18	<6	32	<6	397	803	25	<6	290	638	233
Th1-7	BP	38	<6	71	11	278	2393	142	6	1769	2985	482
Th1-8	BP	n.t.	n.t.	500	156	560	8300	31	58	10300	7100	1200

*pg/ml are shown; cytokine levels in supernatants from T-cell clones (TCC) plus PBMC without allergen were subtracted from levels in supernatants from TCC plus PBMC plus allergen; **Th2 cells produce 10 x IL-4 than IFN-γ; Th1 cells produce 10 x IFN-γ than IL-4; BP, birch pollen extract

A

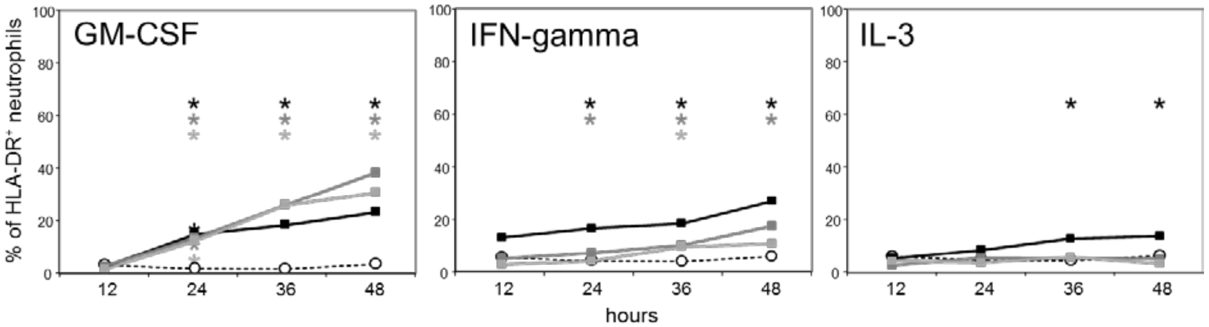

B

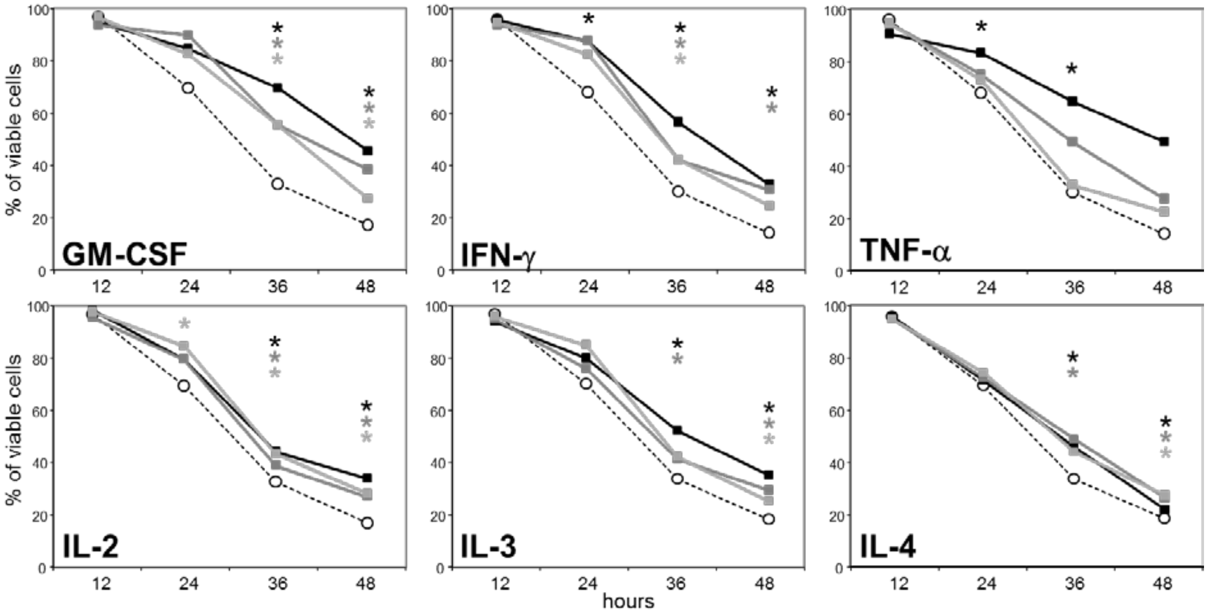

Figure E1. Effects of cytokines on HLA-DR expression and viability of neutrophils from allergic individuals. Neutrophils were kept in medium alone (dotted line) or incubated with recombinant cytokines at a concentration of 10 ng (black line), 1 ng (dark gray), or 0.1 ng (light gray). HLA-DR expression (A) and cell viability (B) were assessed over time by flow cytometry. Squares represent median values of 8 different birch pollen-allergic individuals. *P<0.05, Wilcoxon Signed Ranks test.

Birch pollen-allergic individuals

Birch pollen-allergic patients showed rhinoconjunctivitis in spring, positive skin prick tests to birch pollen extract (ALK-ABELLO, Hørsholm, Denmark) and birch pollen-specific IgE of >0.35 kU_A/l (ImmunoCAP; Thermo Fisher Scientific, Uppsala, Sweden). The study was approved by the ethics committees of the Medical University of Vienna (EK-1344/2018) and conducted in accordance with the Declaration of Helsinki. Patients gave written informed consent.

Isolation and stimulation of neutrophils

Neutrophils were isolated from heparizined peripheral blood by density gradient centrifugation using Ficoll-Hypaque (Pharmacia Diagnostics, Uppsala, Sweden), dextran sedimentation (4% dextran T 500, Carl ROTH, Germany), and osmotic lysis of erythrocytes. The resulting cell population was analyzed by flow cytometry performed with a FACS Canto II using FACS Diva (BD Biosciences, San Jose, CA, USA) and FlowJo software (TreeStar, Inc., Ashland, OR, USA) employing PE-CD14, PerCP-CD16 (3G8), APC-CCR3, BV421-HLA-DR (L243), BV510-CD3, PE-CD19, FITC-CD66b, PE-CD80 and PE-CD86 antibodies (all BD Bio-sciences, San Jose, CA, USA). Viability was determined with the Fixable Viability Dye eFluor® 780 (eBioscience Inc.). Cells ($1x10^6$) were cultured in 500 µl of RPMI 1640 supplemented with 10% autologous plasma without or with recombinant cytokines (Peprotech, Rocky Hill, NJ, USA) at concentration ranges of 0.01-10 ng/ml for up to 48 h. In some experiments, cytokine cocktails corresponding to Th1-1, Th1-2, Th1-3, Th2-1, Th2-2 and Th2-3 (Table E1) were used. For internalization experiments, pHrodo-labelled Bos d 5 (10 µg/ml) was added. In adherence to the 'Guidelines for the use of flow cytometry and cell sorting in immunological studies' the gating strategy for the analysis of neutrophils is shown in Figure E2.

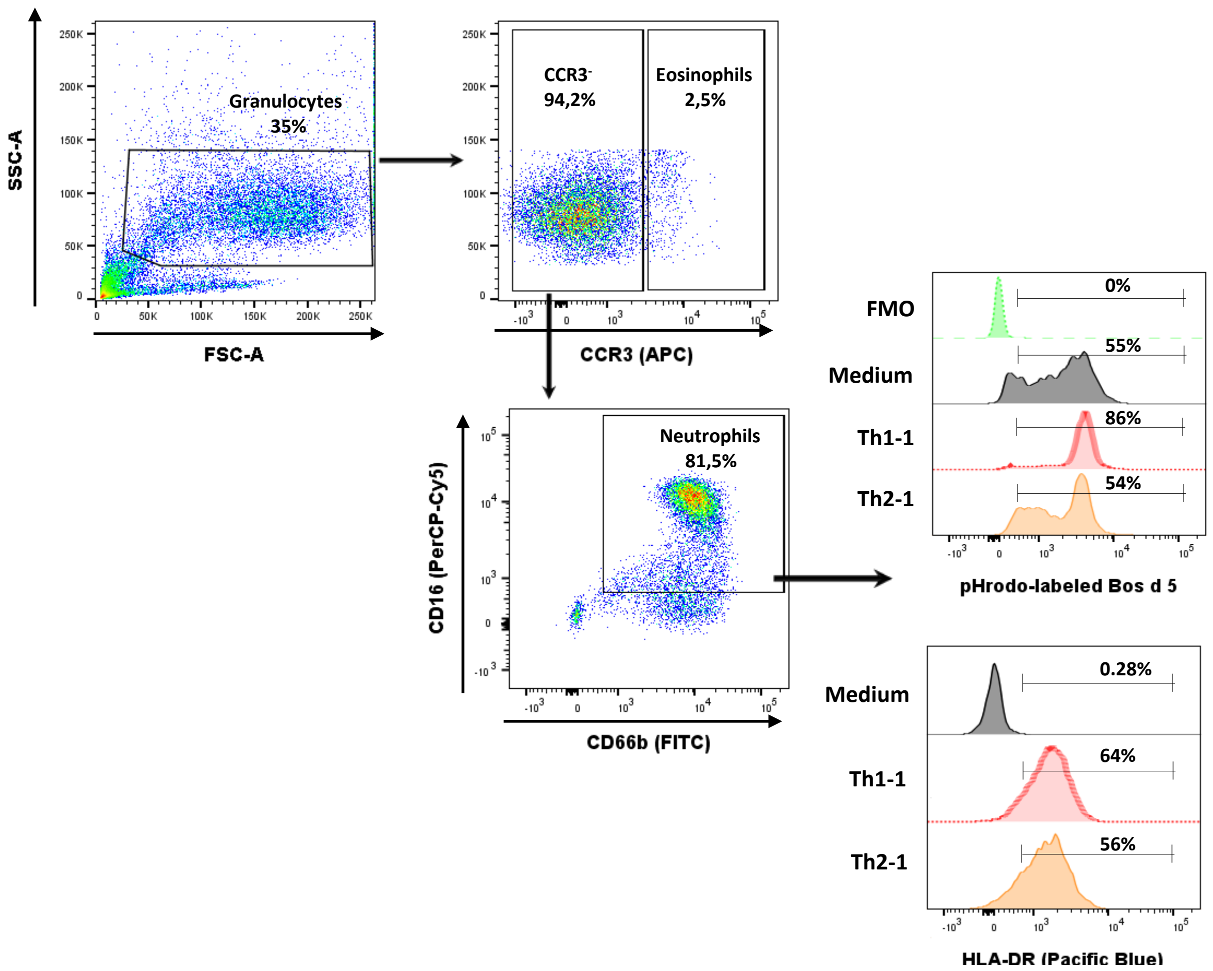

Figure E2. **Characterization of neutrophils**. Cells were cultured for 20 h in medium in the absence or presence of pHrodo-labelled Bos d 5 and the cytokine cocktails Th1-1 and Th2-1. Viable granulocytes were defined by SSC-A/FSC-A properties. Eosinophils were excluded from the population based on CCR3 expression. Neutrophils were identified as $CD66b^{+}CD16^{+}$ cells and the percentage of $pHrodo^{+}$ and $HLA\text{-}DR^{+}$ cells in response to the different stimuli was assessed. The Fluorescence Minus One (FMO) control was used in cultures incubated in medium without pHrodo-labelled Bos d 5.

Stimulation of Bet v 1-specific T cells

Before being used as antigen-presenting cells, neutrophils were applied to an immunomagnetic negative selection step using the human MACSxpress® Neutrophil Isolation Kit (Miltenyi Biotec GmbH, Bergisch-Gladbach, Germany) [1]. This approach resulted repeatedly in >99% pure $CD16^{+}CD66b^{+}CCR3^{-}HLA\text{-}DR^{-}CD19^{-}CD3^{-}CD14^{-}$ cells.

Allergen-specific T cell clones (TCC) were stimulated with autologous PBMC in the absence or presence of allergen (5 µg/ml). After 24 h, supernatants were harvested and cytokines were assessed by bead array (Luminex, Austin, TX, USA). Allergen-specific T-cell lines were expanded from PBMC of birch pollen-allergic patients stimulated with recombinant Bet v 1.0101 (Bet v 1) purchased from Biomay AG, Vienna, Austria as described [2]. Bet v 1-reactive T cells ($2\text{-}5\times10^{4}$, viability >85% as assessed with Bürker chamber of trypan blue-stained cells) were stimulated with purified autologous neutrophils ($5x10^{4}$) that had been cultured without or with Bet v 1 or Bos d 5 (5 µg/ml each) in the absence or presence of the different cytokine cocktails for 24 h, washed three times with PBS, and irradiated with 60 Gray. All experiments were performed in duplicate. After 48 h proliferative responses were measured by ^{3}[H]thymidine incorporation during 16 h as counts per minute (cpm).

Statistics

Data were analyzed using SPSS 20.0 (SPSS, Chicago, IL, USA). Statistical differences were determined by Wilcoxon signed ranks test and considered statistically significant for $P<0.05$.

REFERENCES

1 **Polak, D., Hafner, C., Briza, P., Kitzmuller, C., Elbe-Burger, A., Samadi, N., Gschwandtner, M., Pfutzner, W., Zlabinger, G. J., Jahn-Schmid, B. and Bohle, B.,** A novel role for neutrophils in IgE-mediated allergy: Evidence for antigen presentation in late-phase reactions. *J Allergy Clin Immunol* 2019.**143**:1143-1152 e4.

2 **Kitzmuller, C., Kalser, J., Mutschlechner, S., Hauser, M., Zlabinger, G. J., Ferreira, F. and Bohle, B.,** Fusion proteins of flagellin and the major birch pollen allergen Bet v 1 show enhanced immunogenicity, reduced allergenicity, and intrinsic adjuvanticity. *J Allergy Clin Immunol* 2018. **141**: 293-299 e296.
